# Importance of Microbiome of Fecal Samples Obtained from Adolescents with Different Weight Conditions on Resistance Gene Transfer

**DOI:** 10.3390/microorganisms10101995

**Published:** 2022-10-09

**Authors:** Armando Navarro, Gerardo E. Rodea, Hugo G. Castelán-Sánchez, Héctor Armando Saucedo-Pastrana, Delia Licona-Moreno, Carlos Eslava-Campos, Laura L. Tirado-Gómez, Ariel Vilchis-Reyes, Guadalupe García de la Torre, Verónica Cruz-Licea

**Affiliations:** 1 Public Health Department, Faculty of Medicine, National Autonomous University of Mexico, Av. Universidad 3000, Ciudad Universitaria, Mexico City 04510, Mexico; 2 Bacterial Pathogenicity Laboratory, Hemato-Oncology Research Unit, Federico Gómez Children’s Hospital of Mexico/Faculty of Medicine UNAM, Mexico City 06720, Mexico; 3 Researchers for Mexico Program, Genomics and Evolutionary Dynamics Group of Emerging Microorganisms, National Council of Science and Technology, (CONACYT), Mexico City 82996, Mexico; 4 Public Health Services of Mexico City, Mexico City 04730, Mexico; 5 Peripheral Unit of Basic and Clinical Research in Infectious Diseases, Hemato-Oncology and Research Building, Children’s Hospital of Mexico Federico Gómez, Dr. Márquez 162, Col. De los Doctores, Mexico City 06720, Mexico

**Keywords:** body mass index, resistance genes, microbiome, gut microbiota

## Abstract

Antimicrobial resistance (AMR) is a relevant public health problem worldwide, and microbiome bacteria may contribute to the horizontal gene transfer associated with antimicrobial resistance. The microbiome of fecal samples from Mexican adolescents were analyzed and correlated with eating habits, and the presence of AMR genes on bacteria in the microbiome was evaluated. Fecal samples from adolescents were collected and processed to extract genomic DNA. An Illumina HiSeq 1500 system was used to determine resistance genes and the microbiome of adolescents through the amplification of gene resistance and the V3–V4 regions of RNA, respectively. Analysis of the microbiome from fecal samples taken from 18 obese, overweight, and normal-weight adolescents revealed that the Firmicutes was the most frequent phylum, followed by Bacteroidetes, Actinobacteria, Proteobacteria and Verrucomicrobia. The following species were detected as the most frequent in the samples: *F. prausnitzii*, *P. cori*, *B. adolescentis*, *E. coli* and *A. muciniphila*. The presence of *Bacteroides*, *Prevotella* and *Ruminococcus* was used to establish the enterotype; enterotype 1 was more common in women and enterotype 2 was more common in men. Twenty-nine AMR genes were found for β-lactamases, fluoroquinolones, aminoglycosides, macrolide, lincosamides, streptogramin (MLS), tetracyclines and sulfonamides. The presence of microorganisms in fecal samples that harbor AMR genes that work against antimicrobials frequently used for the treatment of microbial infections such as b-lactams, macrolides, aminoglycosides, MLS, and tetracyclines is of great concern, as these organisms may be an important reservoir for horizontal AMR gene transfer.

## 1. Introduction

Despite efforts to contain the surge of antimicrobial-resistant microorganisms, antimicrobial resistance (AMR) is a public health problem worldwide [1]. The current level of antimicrobial resistance has been recognized as a serious problem, especially because there is still no effective strategy to control the transmission of resistance genes or the rise of resistant strains. Reports show that the prevalence of resistance to antibiotics will continue to rise if no effective new treatments are implemented. This problem will affect 10 million patients per year by 2050 due to the increase in infections by microorganisms resistant to antimicrobials [2]. According to an analysis by collaborators in antimicrobial resistance, infections caused by AMR bacteria led to 1.27 million deaths. Lower respiratory tract infections are among the main causes of death, and six important pathogens have been registered as key AMR bacteria: *Escherichia coli*, *Staphylococcus aureus*, *Klebsiella pneumoniae*, *Streptococcus pneumoniae*, *Acinetobacter baumannii* and *Pseudomonas aeruginosa* [1].

In recent years, it has been estimated that 700,000 people have died due to AMR bacteria worldwide. In Europe, around 33,000 deaths per year are attributed to AMR bacteria [2,3], while in the United States of America (USA) in 2005, methicillin-resistant *S*. *aureus* (MRSA) infections were associated with 94,360 invasive infections and with a record of 18,650 deaths [4]. In 2019, the Centers for Disease Control reported 2.8 million infections by AMR microorganisms and more than 35,000 deaths [5]. The report also stated that infections resistant to antimicrobials are mainly related to 18 microorganisms among which *Clostridium difficile* is prominent and was associated with 223,900 cases and 12,800 deaths. Other reported AMR microorganisms are the extended-spectrum β-lactamase (ESBL)-producing Enterobacteriaceae. A study conducted in a tertiary hospital in Hefei Anhui province, China, reported isolates from clinical samples of *K*. *pneumoniae*, *E*. *coli*, *Enterobacter* *cloacae* and *Serratia marcescens* ESBL strains, carriers of *mcr-1* (colistin) and carbapenemase genes [6]. Similarly, in Peshawar, Pakistan, it was reported *K*. *pneumoniae* produced ESBL from urine samples harboring New Delhi β-lactamase (*bla*_NDM-1_) genes; these microorganisms are classified as serious threats to human health [7].

The situation in Mexico is similar to that in other regions worldwide, with increasing resistance to pathogenic microorganisms associated with community and intrahospital infections [8,9]. This was shown in a study conducted in 14 hospitals by the university health research program at the National Autonomous University of Mexico, in which 11,900 cultures were isolated from urine samples (73%) and blood samples (27%) between 2016 and 2017. The results showed that 91% of bacterial cultures from urine samples were *E. coli*; the remainder were *K. pneumoniae, E. cloacae*, *P. aeruginosa* and *A. baumannii* which were mainly resistant to cephalosporins and ESBL [10]. Other authors analyzed AMR in 22,943 cultures obtained in 2018 from 47 hospitals in Mexico and reported the presence of Gram-negative bacteria resistant to carbapenemases. Phenotypic identification of the cultures showed the presence of *A*. *baumannii*, *P*. *aeruginosa* and *Klebsiella* spp., which were multi-resistant to antimicrobials at frequencies of 50%, 40% and 12%, respectively [9]. Studies analyzing resistance patterns in *E. coli* cultures isolated from urine samples of pregnant and non-pregnant women in two Mexican States (Sonora and Puebla) between April 2017 and December 2018 showed that 92.7% had multidrug resistance (MDR), 6.7% were extensively drug resistant (XDR) and 0.6% were pandrug resistant (PDR) [11].

Next-generation sequencing (NGS) underscores the idea that human diversity depends not only on DNA content, but also on the composition of the microbiome and shows how individual lifestyle influences the shaping of the personal microbiome. The nature of the microbiome contributes not only to health but also to pathogenic status by harboring and exchanging resistance genes [12]. Recent advances in DNA sequencing technology have opened the possibility of monitoring the epidemiology of pathogens harboring AMR genes using NGS [13]. Furthermore, extensive studies of the gut microbiome of human populations with different compositions have shown that the gut microbiota of individuals with obesity is dominated by bacteria from the Firmicutes phylum, whereas Bacteroidetes are most abundant in lean individuals [14]. However, other studies show that diet plays an important role in the composition of the gut microbiome. For example, it has been reported that, in industrialized populations with lower diversity in the microbiota, bacteria of the genus *Bacteroides* dominate, while in hunter-gatherer populations bacteria of the genus *Prevotella* predominate, leading to the assumption that these differences are related to lifestyle and a diet high in fat and fiber [12,15].

The emergence of AMR bacteria suggests comprehensive monitoring of the propagation of pathogenic strains and phenotypic characterization of bacteria is not sufficient. Whole genome sequencing and bacterial rRNA sequencing have made it possible to evaluate the bacterial microbiome from different human samples and detect antimicrobial resistant genes (ARGs) [16,17]. These methods have identified resistance genes from strictly anerobic microorganisms that could not be detected by conventional microbiological techniques [16]. The following study uses NGS of 16S rRNA to detect ARGs and to identify the microbiome of fecal samples from adolescents and the relationship between the family sociodemographic history, health, physical activity, hygiene practices, diet and the body mass index (BMI) of adolescents.

## 2. Materials and Methods

### 2.1. Design and Study Population

This was a transversal observational study based on 18 adolescent fecal samples of both sexes enrolled for the 2019–2020 school year in a public secondary school in the Coyoacán district in southern Mexico City.

### 2.2. Selection Criteria

All adolescents in the school were invited to participate and those who presented a consent form from their parent or legal guardian were accepted. Those who mentioned taking antibiotics, probiotics or any other medicine in the two months before the start of the study start were excluded. Those who submitted liquid stool samples (diarrhea) or did not provide enough samples for analysis were excluded, as were those who did not answer the questionnaire correctly or left out information.

### 2.3. Receipt of Information and Biological Material

A questionnaire was designed to obtain general information relating to health and eating habits from all adolescents with the assistance of their parents. A total 75 multiple-choice questions were asked to obtain information on sex, age, family medical history (obesity, hypertension, diabetes, renal disease), health (diarrhea) and physical activity (Table S1). In addition, questions about weight (kilograms) and height (centimeters) provided data for calculating body mass index (BMI) using the formula weight divided by height squared and for classifying nutritional status: underweight, normal weight, overweight and obese.

The adolescents were asked to provide a stool sample. The adolescents and their parents/guardians were given instructions on how to collect the sample in a sterile plastic container on the first voiding of feces of the day. The samples were transported and maintained in a cooler at 4 °C before DNA collection. This study was conducted in accordance with the principles of the Declaration of Helsinki of the World Medical Association and in accordance with the recommendations of the Ethics and Research Committee of the Faculty of Medicine, National Autonomous University of Mexico, project number FM/DI/056/2018.

### 2.4. Genomic DNA Extraction from the Fecal Samples

The fecal samples were transported and conserved in tubes with buffer solution (DNA/RNA Shield, Zymo Research, R1101-1, Irvine, CA, USA) and kept refrigerated at 4 °C until the DNA extraction process.

### 2.5. Ribosomal 16s Gene Sequencing

The genomic library was created by amplifying the V3–V4 regions of the ribosomal RNA using a paired-end (300 pb) Quick-16S Next-Generation Sequencing kit (NGS Cat. No. D6400, Zymo Research Corp., Irvine, CA, USA) and then sequenced using Illumina HiSeq 1500 through the ZymoBIOMICS metagenomic sequencing service (Zymo Research, Irvine, CA, USA).

### 2.6. Analysis of Antimicrobial Resistance

The ARGs were detected after DNA extraction with combinations of specific primers (primers were the property of Pangea Laboratory) for different classes of antibiotics: carbapenems, cephalosporins, penicillins, aminoglycosides, fluoroquinolones, macrolide, lincosamides, streptogramin (MLS), glycopeptides, tetracyclines, sulfonamides and rifamycins. The Illumina platform was used for sequencing through the PrecisionBIOME service, which detects the most frequent resistance genes and the mutations that confer resistance. The laboratory of Pangea indicated that the AMR genes determined with their system were collected and curated from sequences published in NCBI. Pangea Laboratories provided a database called miDOG of the results of the AMR detected. With this database, analysis of the AMR genes was carried out. A list of genes detected in the fecal samples is attached (Table S2). The comprehensive antibiotic resistance database (CARD) was used for ARGs analysis analysis (https://card.mcmaster.ca/, last access date 28 September 2022).

### 2.7. Bioinformatic Analysis

The FastQC program was used to control the quality of reads and to filter and trim sequences with poor quality; then, DADA2 was used to perform the taxonomic assignment of the reads [18]. The diversity was analyzed using the phyloseq library, and graphs of relative abundance were generated using the ggplot2 library in R. Then, the relative abundances of the genera were determined based on the total number of sequences in each sample [19].

### 2.8. Statistical Analysis

Data were collected and analyzed using the SPSS program (Statistical Package for the Social Science 20.0 software for Windows). Initially, the data were described using the summary measures for the variable type. Later, bivariate analysis was performed to determine the association between each microorganism and sociodemographic data, family medical history, physical activity, hygiene habits, diet and nutritional status. The comparison between sex and nutritional status was made at 95% statistical significance (*p* ≤ 0.05). The odds ratio (OR) was applied with 95% confidence intervals (*p* ≤ 0.05).

## 3. Results

### 3.1. Adolescent Cohort

The cohort included 18 adolescents from whom we analyzed fecal samples focusing on 16S metabarcoding and searching for resistance genes. The adolescents had a minimum age of 10.45 years and a maximum age of 15.39 years, with a median average of 12.79 years. Regarding gender, 11 (61.1%) of the adolescents were girls and 7 (38.9%) were boys. When we looked at BMI, 10 (55.5%) were of normal weight, 5 (27.8%) were overweight and 3 (16.7%) were obese.

### 3.2. Microbiome

**Figure 1:**
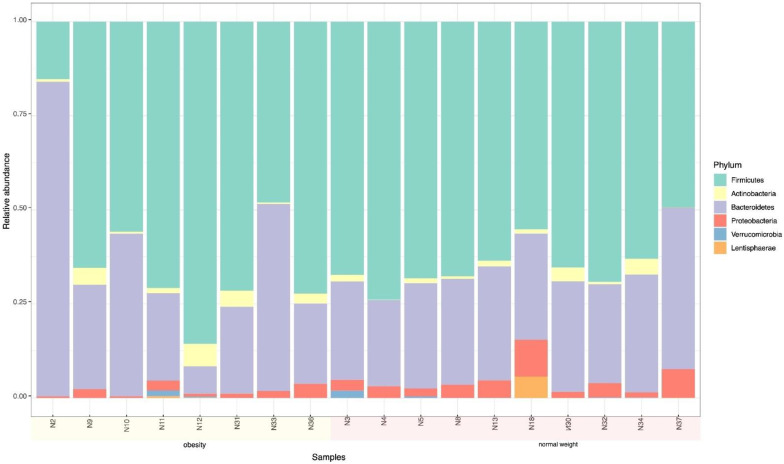
Relative abundance by phylum in adolescent fecal samples. Firmicutes was the most abundant phylum followed by Bacteroidetes, Actinobacteria, Proteobacteria and Verrucomicrobia.

The phylum Firmicutes was predominant in all adolescents followed by Bacteroidetes. Actinobacteria was the next phylum in terms of frequency with the remaining phyla, such as Proteobacteria and Verrucomicrobia, showing low frequency (Figure 1).

Regarding the nutritional status of the adolescents, Bacteroidetes and Firmicutes were the most abundant, 38.3% and 49.4%, respectively. Considering these two phyla in relation to BMI, the frequencies of Bacteroidetes and Firmicutes in the overweight and obese adolescents were 43.38% and 42.13%, respectively, while these frequencies in normal-weight adolescents were 33.84% and 55.41%, respectively (Table 1). From a statistical point of view, the difference in the frequency of Firmicutes of overweight/obese adolescents compared to normal weight adolescents was significant (*p* < 0.05).

### 3.3. Microbial Diversity

Analysis revealed 188 species, of which 115 were specifically identified by name, while the remaining 73 species could not be identified. Regarding the species frequency, 21 species accounted for more than 50% of the species identified, and these were grouped into five taxa. The most abundant species were: *Faecalibacterium prausnitzii* (Firmicutes), *Prevotella copri* (Bacteroidetes), *Bifidobacterium adolescentis* (Actinobacteria), *Escherichia coli* (Proteobacteria) and *Akkermansia muciniphila* (Verrucomicrobia), which in combination represented 50.3% and 47.4% of species in normal weight adolescents and overweight/obese adolescents, respectively (Table 2). At the genus level, 70 genera were identified.

**Figure 2:**
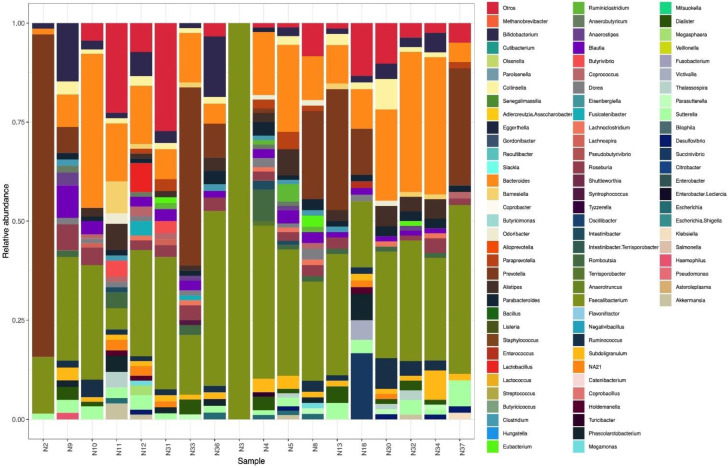
Genera diversity in DNA samples from adolescent fecal samples. The genus *Faecalibacterium* was found in all samples but was not detected as a predominant bacterial genus in any of the three groups of adolescents of the study.

Of these, the phylum Firmicutes was represented by 36 (52.9%) genera, followed by Actinobacteria with 10 (14.7%) genera, Bacteroidetes 10 (14.7%) genera, Proteobacteria 9 (13.2%) genera, and Lentisphaerae 1 (1.5%), Verrucomicrobia 1 (1.5%) and Archaea 1 (1.5%) genus (Figure 2). At the family level, 37 bacterial families were identified with the three most abundant families being Ruminococcaceae (31.3%), Lachnospiraceae (19.24%) and Prevotellaceae (18.3%). Other selected families with lower abundance were Bacteroidaceae (11.9%) and Bifidobacteriaceae (1.8%).

**Figure 3:**
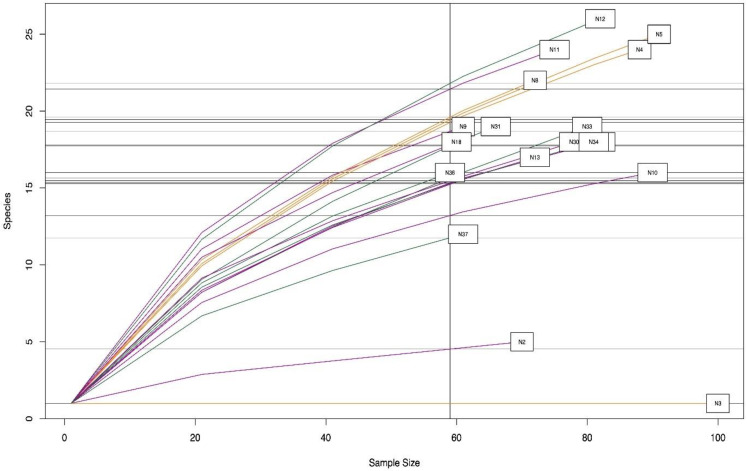
Species accumulation curve in fecal samples. The samples showed asymptomatic behavior leading to similar diversity between samples except for sample N3.

The microbial diversity of each sample was also analyzed, and the corresponding rarefaction curves were plotted (Figure 3). Figure 3 shows the total number of operational taxonomic units (OTUs) as a function of the size of each sample. The curves quickly reach asymptomatic levels for all the adolescent samples. However, sample N3 showed low diversity compared to the other samples, especially samples N11, N18 and N5, which had the highest diversity of genera.

**Figure 4:**
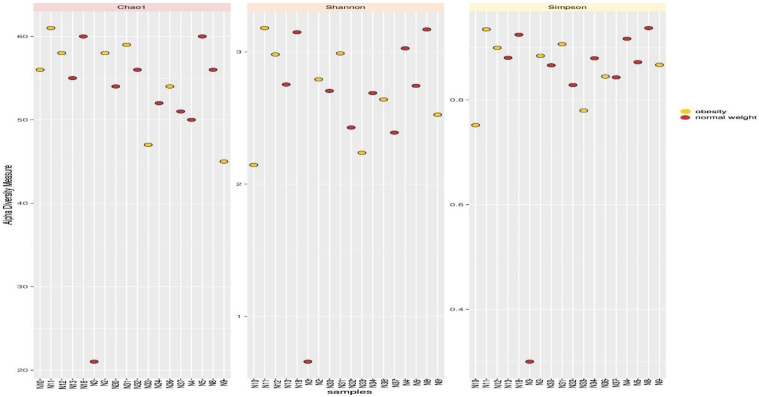
Chao, Shannon and Simpson indexes used to evaluate alpha diversity of the genera. Sample N3 showed more diversity compared to the other samples.

Similarities and differences in microbial composition according to the alpha diversity of the genera were measured using alpha diversity metrics (Chao, Simpson and Shannon). The Chao index represents the richness of the samples and the results from the rarefication curve showed that samples N11, N18 and N5 were the richest in diversity, while sample N3 was the least rich (Figure 4). The Simpson and Shannon indices provide more information about the composition of the samples rather than the richness. The results showed that there were no differences between the diversity of genera among overweight/obese adolescents compared to normal weight adolescents. Once again, these two indices showed that sample N3 had the least diversity.

**Figure 5:**
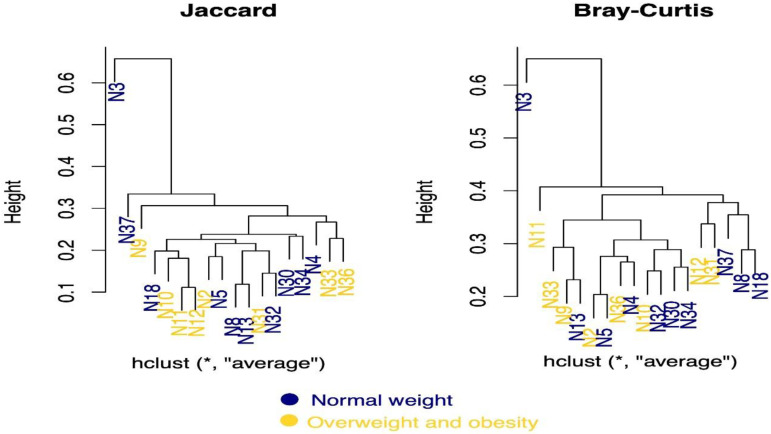
Beta-diversity dendrograms. These show the average distance and clustering of the individual samples according to the Jaccard and Bray-Curtis distance methods. The samples in color blue correspond to normal weight, in yellow color correspond overweight and obese adolescents.

Beta diversity of the samples was then assessed using the Jaccard and Bray–Curtis indices, which quantify the overall taxonomic composition between samples. The Jaccard index ignores exact abundances and only considers presence–absence values, while the Bray–Curtis index considers absences. The results are presented in Figure 5 as a dendrogram showing clusters regarding adolescent weight. Sample N3 was the most deviant and separated from the main cluster. Regardless of the index, samples N30 and N34, corresponding to obese adolescents, were grouped, indicating that both samples had similar bacterial genera. Meanwhile, samples N10, N11 and N12, corresponding to normal-weight adolescents, were grouped according to the Jaccard index, indicating the presence of similar bacteria. However, as mentioned above, the groups in the main cluster did not show strong separation according to BMI status, indicating no significant difference in microbial diversity.

### 3.4. Presence of Beneficial and Pathogenic Bacteria in the Intestinal Microbiota in Adolescents

Bivariate analysis of the metagenome was carried out, and bacteria with beneficiary functions, as well as pathogens against the host, were identified. In terms of beneficial bacteria, *F*. *prausnitzii*, *B*. *longum, P*. *copri* and *B*. *adolescentis* were the three most frequent of the 17 bacteria (Table S3). In contrast, *Collinsella aerofaciens* (Actinobacteria) and *E. coli* (Proteobacteria) were identified as pathogenic bacteria.

*C. aerofaciens* was found more frequently in adolescents who had suffered from diarrhea in the three months prior to this study beginning (Table 3). This pathogen was also related to adolescents with a family history of renal disease (X^2^ = 7.9; *p* = 0.005).

Interestingly, *E. coli* was more frequent in adolescents who performed physical activities (Table 4), in adolescents who presented tooth caries (X^2^ = 4.0; *p* = 0.46) and, although not significantly different, in adolescents who ate a daily breakfast (X^2^ = 7.615; *p* = 0.55).

### 3.5. Enterotypes

Comparing the presence of *Bacteroides* (enterotype 1), *Prevotella* (enterotype 2) and *Ruminococcus* (enterotype 3) in the adolescents, frequencies of 47.6%, 49.6% and 3.3%, respectively, were recorded. In terms of enterotypes by sex, enterotype 1 was more frequent in the adolescent girls (Table 5) and in those adolescents whose families did not exhibit obesity compared to those that did exhibit obesity in their family members (X^2^ = 5.9, *p* = 0.0015). Applying these same two parameters to enterotype 2, this enterotype was found to be more frequent in the adolescent boys X^2^ = 7.901, *p* = 0.005 (Table 5), and in those adolescents whose family members exhibited obesity X^2^ = 5.951, *p* = 0.015.

### 3.6. Presence of Resistance Genes to Antimicrobials

**Figure 6:**
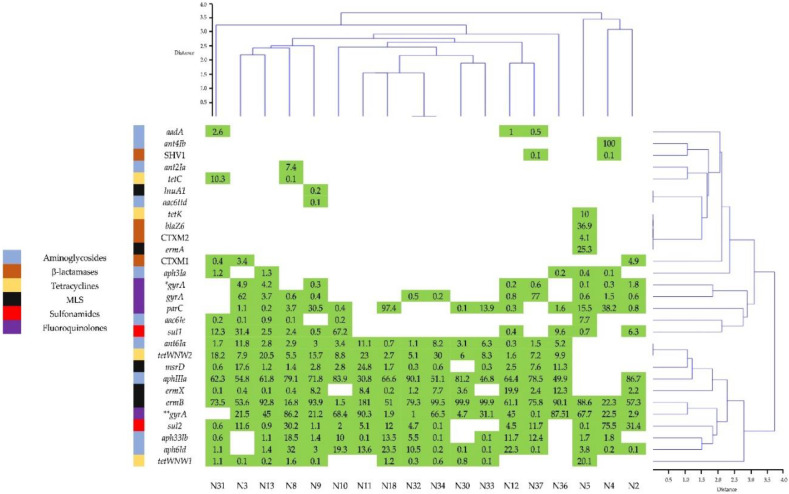
Presence of AMR genes in adolescent fecal samples. The numbers shaded in green indicate the presence of the particular gene, and the area in white indicates the absence of a gene. The samples and AMR genes are clustered in a similar way. * *gyrA* gene belong to the 259AAC allele, and ** *gyrA* belong to 247 TTG allele.

Figure 6 and Table S4 show the results of the AMR gene analysis in the 18 fecal samples, which detected 29 AMR genes belonging to aminoglycosides, β-lactamases, tetracyclines, MLS, sulfonamides and fluoroquinolones. Each sample presented a different pattern of AMR genes, and the samples self-grouped according to hierarchical clustering. Samples N5, N4 and N2 formed a group with a resistance-gene pattern different from the other samples (Figure 6).

The more frequent genes were *ermB* (100%), *gyrA E. coli* (94%), *aph3IIIa*, (88.8%), *aphId* (88.8%), *tetWNW2* (83.3%), *aph33Ib* (77.7%), *msrD* (77.7%) and *ermX* (77.7%) (Figure 6). The average expression of the most frequent genes were *ermB* (74.3%), *gyrA E. coli* (36.9%) and *aph3III* (59.0%). Turning to antimicrobial resistance genes, enterobacteria *K*. *oxytoca* and *E*. *coli* presented genes against penicillins, cephalosporins, lincosamides, aminoglycosides and sulfonamides (Table S5). Other microorganisms were also analyzed, and, in the case of *C*. *areofaciens*, genes resistant to doxycycline, tetracycline and minocycline were found in 14 (77.8%) of the DNA samples. *F*. *prausnitzii* was found in all 18 (100%) of the samples, as were genes resistant to lincosamides, macrolides and tetracyclines, while genes resistant to aminoglycosides were found in only 3 (16.7%) of the samples.

## 4. Discussion

Humans have coevolved with trillions of microbes that inhabit their bodies, forming ecosystems and habitat-specific complexes that are finely tuned to a highly changing physiology [20]. In this study described above, the taxonomic diversity and the presence of resistance genes of the microbiota in fecal samples from adolescent Mexicans with different BMI were analyzed.

A total of 70 genera were identified, belonging mainly to the phylum Firmicutes, followed by Actinobacteria, Bacteroidetes, Proteobacteria, Fusobacteria, Lentisphaerae, Tenericutes, Verrucomicrobia and, to a lesser extent, Melainabacteria and Euryarchaeota (Archaea). López-Contreras [21] carried out an analysis of genera in fecal samples from children aged between 6 and 12 years with normal weight and obesity and found no difference in the amount of diversity in the Bacteroidetes and Firmicutes genera. In the current study, the most abundant genera belonged to the phylum Firmicutes (62 genera).

As mentioned earlier, Firmicutes was much more abundant than Bacteroidetes in normal weight adolescents. The results described in this study differ from previous publications in which the authors reported that the microbiota of obese individuals was dominated by Firmicutes, whereas Bacteroidetes was more abundant in thinner individuals [14]. Similarly, Méndez-Salazar [22] studied children age 9 to 11 years of age from public schools in the State of Mexico who were either obese or malnourished and found little bacterial diversity when compared to normal-weight children.

Concerning OTUs, the curves climbed rapidly in an asymptomatic way for all the adolescent samples. However, sample N3 showed lower diversity compared with other samples, such as N5, N11 and N18, which exhibited a large diversity of genera. Sample N3 corresponded to an adolescent of normal weight in which *F. prausnitzii* was detected. Interestingly, samples N5 and N11 also corresponded to adolescents with normal weight, while N18 came from an overweight adolescent. *F. prausnitzii* relates to a typical Mediterranean diet that is high in complex carbohydrates and provides protective benefits against metabolic diseases, such as diabetes and other metabolic disorders [23]. Other studies have reported that normal weight individuals have a more abundant diversity of species than obese and underweight individuals [22,24]. In the current study, no difference was found in species diversity within samples from the three BMI categories.

The Chao, Simpson and Shannon alpha diversity analysis revealed no differences in genera diversity between obese and normal weight adolescents. The Chao index confirmed the OTU results in identifying sample N3 as less diverse. The Chao, Simpson and Shannon indices showed no significant difference in genera diversity in the three BMI conditions, which concur with the results reported by López-Contreras [21].

*F*. *prausnitzii*, *B*. *longum, P*. *copri* and *B*. *adolescentis* were the most abundant of the 17 beneficial bacteria identified in the current study. In fact, *F*. *prausnitzii* was present in all samples irrespective of the BMI. In terms of short-chain fatty acids (SFCAs), *Bacteriodes* and *Faecalibacterium* were found as well as *F*. *prausnitzii*, which has anti-inflammatory properties ([25,26]. Mayorga Reyes [27] conducted a study in Mexico in normal weight, overweight and obese young adults and reported that *B. longum* and *F*. *prausnitzii* were the most frequent microorganisms in thin and overweight young adults, and the study analysis suggested that these microorganisms are related to a fiber-rich diet. Due to the presence and abundance of this bacteria in fecal samples, it may be possible to consider these as markers for anti-inflammatory activity in the population of this current study. However, more research is required to be certain that this bacterium did provide such specific benefit to the cohort.

Another beneficial bacterium identified with greater frequency (12.7%) was *P*. *copri*, which some authors have noted as useful as a probiotic [28]. Additional studies revealed that *P*. *copri* reduces glucose levels in rats and healthy humans, which reinforces the concept that it could be used as a probiotic to prevent obesity and type 2 diabetes [29]. This bacterium needs to be studied in more detail to determine the role it plays in intestinal dysbiosis, as well as in healthy individuals. Turning to another beneficial bacterium, *B*. *adolescentes* was found in 11 of the samples. According to previous report, *B*. *adolescentes* has a beneficial role as a probiotic for its host, and studies attribute the production of gamma aminobutyric acid (GABA) to this bacterium GABA, suggesting that the bacterium plays a role in the modulation of the gut–brain response [30].

Regarding pathogenic bacteria, a variety including *C. aerofaciens* (Actinobacteria) was found in 14 adolescents with greater frequency in those who presented a diarrheal episode in the preceding 3 months of the study. In addition, a relationship between this bacterium and families with a history of renal disease was shown. There have been numerous studies related to *C. aerofaciens*. Nirmalkar [31] analyzed fecal samples from Mexican children and adolescents and found that bacteria of the *Collinsella* genus was four times more abundant in obese adolescents than adolescents of normal weight, as well as seeing a relationship between this genus and high levels of cholesterol and triglycerides. In a different study related to *C. aerofaciens* in patients with rheumatoid arthritis, Mena-Vázquez [32] reported an association between age, tobacco use and high levels of anti-cyclic citrullinated peptide (anti-CCP). Further research is required to determine the additional roles that *C. aerofaciens* plays apart from its association with diarrhea and rheumatoid arthritis.

Analysis of the enterotypes showed that enterotype 1 was more frequent in female adolescents and related to adolescents with no obesity among family members. In contrast, enterotype 2 was more frequent in male adolescents. Enterotype 1 is associated with diets rich in protein, while enterotype 2 is associated with diets rich in carbohydrates [33]. Maya-Luca [34] studied 9- to 11-year-olds in Mexico City with normal weight and obesity. The study results reported that the normal weight children had an abundance of *Ruminococcus* spp. (enterotype 3), while the obese children had a microbiota dominated by *Prevotella* spp. (enterotype 2). These results differ from those reported here because enterotype 3 was not detected in the samples.

The gastrointestinal (GI) tract is an important reservoir of AMR genes, which are interchanged between the pathogens [35]. In this study, 29 AMR genes belonging to antibiotics most frequently prescribed to outpatients in México, such as b-lactams, aminoglycosides, lincosamides, macrolides, tetracyclines and sulfonamides were detected, as reported previously [8]. The results from the current study did not find *TEM*, while *CTXM2* and *SHV1* were found in low levels, and *CTXM1* was found in 16.7% of samples. Another gene related to β-lactamases was *blaZ6*, which was present in only one sample, although the frequency of this gene varies depending on the region; for example, in Japan, it has a frequency between 1% and 3% [36].

Kareem [37] showed the presence of the *gyrA* gene in *K*. *pneumoniae* strains, which presented resistance to fluoroquinolones at a frequency of 62.8%. The current study found that the *gyrA* gene had a frequency of 94.4%, which was second behind the *ermB* gene, which presented a 100% frequency in all 18 samples. Another gene that was detected was *parC,* which is related to resistance against fluoroquinolones [38]; this gene was detected with a frequency of 72.2%, which falls in the top 10 most frequent genes in the current study.

The results showed that aminoglycosides presented 10 genes, the largest number for all the antimicrobials in the study. The most frequent of these 10 genes were *aph3IIIa* (88.8%), *aph6Id* (88.8%), *ant6Ia* (83.3%) and *aph33Ib* (77.7%). The remaining six genes did not reach 50% frequency. Lim [39] reported the presence of *aph3IIIa* in pig and cattle isolates and other genes that conferred resistance to aminoglycosides. The *aph3IIIa* gene codes a protein that inactivates kanamycin, neomycin and amikacin, and it was found in 16 (88.9%) of the current study samples. Woegerbauer [40] reported frequencies of *aph3IIIa* in clinical isolates of *E*. *coli* (0.47%), *Enterococcus* spp. (37.5%) and *S*. *aureus* (2.9%). Recently, Machado [41] detected 24 genes related to antimicrobials including aminoglycosides in *F*. *prausnitzii*. The results of the current study showed that *F*. *prausnitzii* harbored aminoglycoside genes in three (16.7%) of the samples. With this in mind, *F*. *prausnitzii* could be used as a probiotic since the microorganism lacks AMR genes, making it unlikely to be a reservoir or source of dissemination for AMR genes.

Analysis revealed the presence of *ermB, ermX, ermA, msrD* and *lnuA1* genes, which confer resistance to MLS. The *ermB* gene was the most frequently detected in all 18 (100%) of the samples. Szczuka [42] reported the presence of the *ermB* gene in isolates of *Staphylococcus hominis* from clinical samples resistant to erythromycin.

Regarding resistance to tetracyclines, the results showed the presence of *tetWNW1*, *tetWNW2*, *tetC* and *tetK*, with *tetWNW2* having the highest frequency (83.3%) amongst the genes resistant to tetracyclines. The *tetW* genes have been reported in different species of the *Bifidobacterium* genus, as well as in *Bifidobacterium animalis* subesp. *Lactis* has been used as a probiotic [43,44]. Meanwhile, Smoglica [45] reported the presence of the *tetK* gene in fecal samples from wild and domesticated livestock.

Two sulfonamide genes, *sul1* and *sul2*, were found in 10 (55.6%) and 15 (83.3%) of our 18 samples, respectively. These results compare well with Gerzova [46], which reported *sul1* and *sul2* genes as being the most frequent in fecal and intestinal samples from pigs.

Tavella [47] suggested that AMR genes are mainly harbored in bacterial families present in the microbiome of the GI tract, such as Lachnospireaceae, Ruminococcaceae and Bifidobacteriaceae. In the present study, the bacterial families Ruminococcaceae (31.3%) were found in the largest amounts, Lachnospiraceae in the second largest amounts (19.24%) and Bifidobacteriaceae in eighth place (1.8%) of the 37 families identified in the analyzed samples. The current study suggests that, as reported by Tavella, the AMR genes identified in the fecal samples from adolescents could be harbored in the germs of the mentioned bacterial families [47].

## 5. Conclusions

Sequencing analysis of the intestinal microbiota of healthy adolescents revealed essential differences. There was a diversity of microbial genera, with Firmicutes having the highest prevalence in normal-weight adolescents. Differences in enterotypes were also detected, showing enterotype 1 as the most prevalent in female adolescents and enterotype 2 the most frequent in male adolescents without considering BMI. This study identified the vast diversity of AMR genes in fecal samples from healthy adolescents, which shows that antimicrobials must be restricted to avoid a surge in multi-resistant microorganisms and their dissemination within the population. Further studies are needed to examine the presence of microorganisms, such as *C*. *aerofaciens* and *E*. *coli*, in healthy adolescents.

## Figures and Tables

**Table 1:** Frequency of bacterial taxonomic groups in adolescents with different BMI status.

Phylum	W + O + N	W + O	N
Relative Frequency %			
None; Others	4.4	5.88	3.32
* Euryarchaeota	0.0	0.00	0.02
Melainabacteria	0.0	0.13	0.20
Actinobacteria	3.9	5.00	2.62
Bacteroidetes	38.3	43.38	33.84
Firmicutes	49.4	42.13	55.41
Lentisphaerae	0.0	0.00	0.33
Proteobacteria	3.3	2.63	3.97
Tenericutes	0.0	0.25	0.07
Verrucomicrobia	0.6	0.63	0.19
Total	99.9	100.0	100.0

* Archaea. O: Obesity; W: Overweight; N: Normal weight; BMI: Body Mass Index

**Table 2:** Most frequent species in DNA samples from adolescents with different BMI status.

Phylum	Species	Normal Weight	Overweight and Obese
Frequency %			
Actinobacteria	*Bifidobacterium adolescentis*	0.20	1.46
	*Collinsella aerofaciens*	1.21	1.00
Bacteroidetes	*Bacteroides stercoris*	0.90	3.46
	*Bacteroides massiliensis*	0.83	0.91
	*Alistipes putredinis*	1.23	0.90
	*Barnesiella intestinihominis*	0.08	1.06
	*Bacteroides dorei*	0.30	1.70
	*Bacteroides vulgatus*	5.21	2.46
	*Prevotella copri*	3.40	12.78
	*Bacteroides uniformis*	1.23	0.55
	*Prevotella stercorea*	2.31	0.00
Firmicutes	*Lactobacillus ruminis*	0.03	0.79
	*Dorea formicigenerans*	0.40	0.35
	*Roseburia faecis*	0.49	1.04
	*Ruminococcus bicirculans*	1.05	0.54
	*Faecalibacterium prausnitzii*	29.37	16.75
Proteobacteria	*Desulfovibrio piger*	0.33	0.13
	*Sutterella wadsworthensis*	1.17	0.86
	*Escherichia coli*	0.40	0.24
Verrucomicrobia	*Akkermansia muciniphila*	0.17	0.45
Total		50.30	47.40

**Table 3:** Relationship between diarrhea and the presence of *C*. *aerofaciens* in adolescents.

		*C*. *aerofaciens*		Total (%)
		Positive	Negative	
Diarrhea	Yes	2	2	4 (22.2)
	No	13	1	14 (77.8)
Total		15	3	18 (100)

X^2^ = 4.1; *p* = 0.04.

**Table 4:** Relationship between the presence of *E*. *coli* and physical activity in adolescents.

		*E*. *coli*		Total (%)
		Positive	Negative	
Physical activity	Yes	8	1	9 (50)
	No	4	5	9 (50)
Total		12	6	18 (100)

X^2^ = 4.0, *p* = 0.046.

**Table 5:** Enterotype by gender in adolescents.

	Enterotype	
Adolescents	1	2
	*Bacteroides* sp.	*Prevotella* sp.
N (%)		
Boys	1 (5.6)	6 (33.3)
Girls	9 (50.0)	2 (11.1)
	10 (55.6)	8 (44.4)

Enterotypes 1 and 2: X^2^ = 7.901, *p* < 0.005.

## Data Availability

Not applicable.

## Supplementary Materials

The following supporting information can be downloaded at: https://www.mdpi.com/article/10.3390/microorganisms10101995/s1, Table S1: Dietary habits and socioeconomic questionnaire, Table S2: List of antimicrobial resistance genes miDOG, Table S3: Species that have a beneficial role for the host, Table S4: AMR genes detected in adolescent fecal samples and Table S5: Presence of resistance genes in *E*. *coli* and *K*. *oxytoca* DNA from adolescent fecal samples.
