# Supplementary material for: Importance of Microbiome of Fecal Samples Obtained from Adolescents with Different Weight Conditions on Resistance Gene Transfer
Source: Microorganisms. 2022 Oct 9;10(10):1995. doi: 10.3390/microorganisms10101995 (PMC9611664; doi:10.3390/microorganisms10101995)
Supplement: Supplementary file 1 [file microorganisms-10-01995-s001.zip › Supplementary tables_Microbiome_A Navarro_28092022.pdf]

Table S3. Species that have a beneficial role for the host.

| Number of Species | Phylum | Species common to the 3 groups (obesity, overweight & normal weight) | Number of samples presenting the species | Frequency % |
|---|---|---|---|---|
| 1 | Verrucomicrobia | *Akkermansia muciniphila* | 1 | 0.90 |
| 2 | Actinobacteria | *Bifidobacterium adolescentis* | 11 | 0.50 |
| 3 | | *Bifidobacterium bifidum* | 7 | 0.20 |
| 4 | | *Bifidobacterium catenulatum* | 7 | 0.20 |
| 5 | | *Bifidobacterium longum* | 12 | 0.40 |
| 6 | | *Bifidobacterium pseudocatenulatum* | 3 | 0.20 |
| 14 | Bacteroidetes | *Prevotella copri* | 9 | 5.30 |
| 7 | Firmicutes | *Blautia luti* | 9 | 0.40 |
| 8 | | *Blautia massiliensis* | 4 | 0.20 |
| 9 | | *Blautia obeum* | 6 | 0.30 |
| 10 | | *Blautia provencensis* | 11 | 0.30 |
| 11 | | *Dorea formicigenerans* | 9 | 0.20 |
| 12 | | *Dorea longicatena* | 6 | 0.20 |
| 13 | | *Faecalibacterium prausnitzii* | 18 | 23.40 |
| 15 | | *Ruminococcus bicirculans* | 5 | 0.50 |
| 16 | | *Ruminococcus faecis* | 2 | 0.10 |
| 17 | Proteobacteria | *Sutterella wadsworthensis* | 11 | 0.70 |

Table S4. AMR genes detected in adolescent fecal samples.

| Frequency of genes | Class | Family | Gene | Number of simples with AMR genes (%) |
|---|---|---|---|---|
| 4 (13.8) | Beta-lactam | ClassA.beta.lactamase | *CTXM2* | 1 (5.6) |
| | | ClassA.beta.lactamase | *CTXM1* | 3 (16.7) |
| | | Staph.blaZ, penicillinase encoded by blaZ | *blaZ6* | 1 (5.6) |
| | | ß-lactamasas plasmídicas: SHV-1, | *SHV1* | 2 (11.1) |
| 4 (13.8) | Fluoroquinolone | *E. coli* gyrA | ***gyrA* | 17 (94.4) |
| | | gyrA Wildtype | *gyrA* WT | 11 (61.1) |
| | | *K.oxytoca* gyrA | ****gyrA* | 11 (61.1%) |
| | | Enterococcus.faecium.parC | *parC* | 13 (72.2) |
| 10 (34.5) | Aminoglycosides | Plasmid or integron-encoded | *ant2Ia* | 1 (5.6) |
| | | aminoglycoside.acetyltransferase | *aac6IId* | 1 (5.6) |
| | | aminoglycoside.acetyltransferase | *ant6Ia* | 15 (83.3) |
| | | aminoglycoside.acetyltransferase | *aac6Ie* | 6 (33.3) |
| | | aminoglycosides.nucleotidyltransferase | *aadA* | 2 (11.1) |
| | | Kanamycin.nucleotidyltransferase | *ant4lb* | 1 (5.6) |
| | | aminoglycoside.phosphotransferase | *aph3Ia* | 5 (27.8) |
| | | aminoglycoside.phosphotransferase | *aphIIIa* | 16 (88.9) |
| | | aminoglycoside.phosphotransferase | *aph33Ib* | 14 (77.8) |
| | | aminoglycoside.phosphotransferase | *aph6Id* | 16 (88.9) |
| 5 (17.2) | MLS* | Erm.23S.ribosomal.rna.methyltransferase | *ermA* | 1 (5.6) |
| | | Erm.23S.ribosomal.rna.methyltransferase | *ermX* | 14 (77.8) |
| | | ribosomal.rna.methyltransferase | *ermB* | 18 (100) |
| | | ABC.F.ribosomal.protection.protein | *msrD* | 14 (77.8) |
| | | lincosamide.nucleotidyltransferase | *InuA1* | 1 (5.6) |
| 4 (13.8) | Tetracycline | efflux.pump | *tetC* | 2 (11.1) |
| | | tetracycline.efflux.pump | *tetK* | 1 (5.6) |
| | | ribosomal.protect.protein | *tetWNW2* | 15 (83.3) |
| | | ribosomal.protect.protein | *tetWNW1* | 11 (61.1%) |
| 2 (6.9) | Sulfonamide | sulfonamide.resistant.dihydropteroate | *sul1* | 10 (55.6) |
| | | sulfonamide.resistant.dihydropteroate.synthase | *sul2* | 15 (83.3) |

* MLS: Macrolides, lincosamides, streptogramines
**gyrA gene belong to the 259AAC allele and
*** *gyrA* belong to 247 TTG allele”

**Table S5.** Presence of resistance genes in *E. coli* and *K. oxytoca* DNA from adolescent fecal samples.

| | | | Penicillins | | | | Cephalosporins | | | | | | | Lincosamides | | Macrolides | | Aminoglycosides | | | sulfonamide | |
|---|---|---|---|---|---|---|---|---|---|---|---|---|---|---|---|---|---|---|---|---|---|---|
| Code | Species | Frequency | AMP | AMX | OXA | PenG | CFR | CFZ | CPD | EFT | CFL | CVN | CAZ | CLIN | MY | ERY | AZM | N | AMK | GM | S | Genes present in the samples |
| N37 | *K. oxytoca* | 0.508 | d | d | d | d | d | d | d | d | d | d | d | - | - | - | - | d | d | d | d | 15 |
| N18 | *E. coli* | 0.427 | - | - | - | - | - | - | - | - | - | - | - | - | - | - | - | d | d | d | d | 4 |
| N10 | | 0.091 | - | - | - | - | - | - | - | - | - | - | - | - | - | - | - | d | d | d | d | 4 |
| N11 | | 0.686 | - | - | - | - | d | d | d | d | d | d | d | - | - | - | - | - | - | - | d | 8 |
| N33 | | 0.151 | d | d | d | d | d | d | d | d | d | d | d | - | - | - | - | d | d | d | d | 15 |
| N4 | | 1.039 | - | - | - | - | - | - | - | - | - | - | - | d | d | d | d | d | d | d | d | 8 |
| N5 | | 0.756 | - | - | - | - | d | d | d | d | d | d | d | - | - | - | - | d | d | d | d | 11 |
| N34 | | 0.263 | - | - | - | - | - | - | - | - | - | - | - | - | - | - | - | d | d | d | d | 4 |
| N36 | | 0.881 | - | - | - | - | - | - | - | - | - | - | - | - | - | - | - | - | - | - | d | 1 |
| N30 | | 0.084 | - | - | - | - | - | - | - | - | - | - | - | - | - | - | - | d | d | d | d | 4 |
| N=18 (%) | | | 2 (11.1) | 2 (11.1) | 2 (11.1) | 2 (11.1) | 4 (22.2) | 4 (22.2) | 4 (22.2) | 4 (22.2) | 4 (22.2) | 4 (22.2) | 4 (22.2) | 1 (5.6) | 1 (5.6) | 1 (5.6) | 1 (5.6) | 8 (44.4) | 8 (44.4) | 8 (44.4) | 10 (55.6) | |

d: Gene detected; -, gene not detected.

AMP, Ampicillin; AMX, Amoxicillin; OXA, Oxacillin; PenG, Benzylpenicillin; CFR, Cefadroxil; CFZ Cefazolin; CPD, Cefpodoxime; EFT, Ceftiofur; CFL, Cephalexin; CVN, Cefovecin; CAZ, Ceftazadime; CLI, Clindamycin; MY, Lincomycin; ERY, Erythromycin; AZM, Azithromycin; N, Neomycin; AMK, Amikacin; GM, Gentamicin; S, Sulfonamide.
