# Supplementary material for: Importance of Microbiome of Fecal Samples Obtained from Adolescents with Different Weight Conditions on Resistance Gene Transfer
Source: Microorganisms. 2022 Oct 9;10(10):1995. doi: 10.3390/microorganisms10101995 (PMC9611664; doi:10.3390/microorganisms10101995)
Supplement: Supplementary file 1 [file microorganisms-10-01995-s001.zip › Table S1. Dietary habits and socioeconomic questionnaire_17092022.pdf]

Universidad Nacional Autónoma de México
Facultad de Medicina
Departamento de Salud Pública

Study of the intestinal microbiota of strict and facultative anaerobes in schoolchildren with different body composition

Folio:………………………………………………….
Application date:……………………………..

Student name: ……………………………………………
Date of Birth ……………………
Group:…………………………………………………..

**Sociodemographic background**			
1	Sex	Boy	Girl
2	Weight		
3	Size		
4	Body Mass Index		
5	Delegation where do you live		
6	mother's education		
7	Father's education		
8	mother's occupation		
9	father's occupation		
**Do you have any of the following at home?**			
10	How many rooms does your house have?		
11	How many levels or floors does your house have?		
12	Drinking water (tap/piped)	Yes	No
13	Light	Yes	No
14	Sewer system	Yes	No
15	Internet	Yes	No
16	Computer equipment	Yes	No
17	Cell phone	Yes	No
18	Cable television	Yes	No
19	Has a car	Yes	No
20	Has own house	Yes	No
**Do any of your family members suffer from any of the following illnesses?**			
21	Mellitus diabetes	Yes	No
22	Arterial hypertension	Yes	No
23	Obesity	Yes	No
24	Cancer	Yes	No
25	Renal disease	Yes	No
26	Tuberculosis	Yes	No
27	Heart disease	Yes	No
**Con respecto a sus hábitos alimenticios**			
28	Number of meals per day		
29	Number of breakfasts per week		
30	Number of dinners per week		
31	Do you have a fixed time to eat?	Yes	No

32	Do you know the plate of good eating?	Yes	No
33	Do you know the pitcher of good drinking?	Yes	No
34	Number of times you eat meat per week		
35	Number of times you eat legumes per week		
36	Number of times you eat whole grains per week		
37	Number of times you eat fruit per week		
38	Number of times you eat vegetables per week		
39	Number of times you drink water per week		
40	Number of times you consume sugary drinks per week		
41	Number of times you eat fried foods per week		
42	Number of times you eat fried or battered foods per week		
43	Number of times you eat cupcakes or cookies per week		
44	Wash fruits and vegetables before eating		
**Regarding your activities, mention**			
45	Do some physical activity or sport?	Yes	No
46	Spend more than an hour in front of the television	Yes	No
47	Have you ever smoked	Yes	No
48	Have you ever consumed an alcoholic beverage?	Yes	No
49	Number of times you bathe per week		
50	Number of times you brush your teeth per day		
51	Number of times you wash your hands per day		
52	Wash your hands after going to the bathroom	Yes	No
53	Wash your hands before eating	Yes	No
54	Use soap when washing hands	Yes	No
55	Has pets?	Yes	No
56	What kind of pet?		
**Regarding your health**			
57	How often do you go to the bathroom (defecate) per day?		
58	Do you suffer from constipation?	Yes	No
59	Has had antibiotic treatment (last 2 months)	Yes	No
60	Has had antiparasitic treatment (last month)	Yes	No
61	Have you had an upset stomach (last month)	Yes	No
62	Suffer from heartburn	Yes	No
63	Has presented diarrhea (last month)	Yes	No
64	Type of stool: Hard, Soft, Liquid		
65	Fever (fever, temperature >38.5) (last month)	Yes	No
66	Are you currently suffering from any illness?	Yes	No
67	Which?	Yes	No
68	Do you have any allergies?	Yes	No
69	How many hours do you sleep?		
70	Do you have a food intolerance?	Yes	No
**Physical exploration**			
71	Acanthosis	Yes	No
72	Gingivitis	Yes	No
73	Cavities	Yes	No
74	Number of cavities		
75	Missing teeth		
